# Group II phospholipase A_2_ in human gingiva with periodontal disease

**DOI:** 10.1155/S0962935195000159

**Published:** 1995-03

**Authors:** H. Shinohara, T. Komatsubara, H. Tojo, M. Okamoto, S. Nishikawa, T. Nagata, H. Ishida

**Affiliations:** 1 Department of Periodontology and Endodontology School of Dentistry the University of Tokushima 3 Kuramoto-cho Tokushima 770 Japan; 2 Department of Molecular Physiological Chemistry Osaka University Medical School 2 Yamadaoka Suita, Osaka 565 Japan

## Abstract

Fourteen kilodalton phospholipase A_2_ molecules (PLA_2_) are classified into two groups, I- and II-PLA_2_, and only the latter has been considered to play a pathogenetic role in various forms of tissue inflammation. Previously we demonstrated high PLA_2_ activity in gingival crevicular fluid (GCF) of patients with periodontal disease, without determining the group of the enzyme involved. In this study, the activity, groups and levels of enzyme in gingiva taken from 13 sites of periodontal disease were determined using both biochemical and radioimmunological methods. A linear correlation between the activity and the level of II-PLA_2_ was observed. No I-PLA_2_ was found in any of the samples tested. These data suggest that the PLA_2_ activity found in the GCF of patients with periodontal disease does not belong to the I-PLA_2_ but to the II-PLA_2_ group.


Mediators of Inflammation 4, 95–97 (1995)

### Group II phospholipase $A_2$ in human gingiva with periodontal disease

H. Shinohara,[1] T. Komatsubara,[2] H. Tojo,[2] M. Okamoto,[2] S. Nishikawa,[1] T. Nagata[1] and H. Ishida[1,CA]

[1]Department of Periodontology and Endodontology, School of Dentistry, the University of Tokushima, 3 Kuramoto-cho, Tokushima 770, Japan; [2]Department of Molecular Physiological Chemistry, Osaka University Medical School, 2 Yamadaoka, Suita, Osaka 565, Japan.

[CA] Corresponding Author

**FOURTEEN kilodalton phospholipase $A_2$ molecules ($PLA_2$) are classified into two groups, I- and II-$PLA_2$, and only the latter has been considered to play a pathogenetic role in various forms of tissue inflammation. Previously we demonstrated high $PLA_2$ activity in gingival crevicular fluid (GCF) of patients with periodontal disease, without determining the group of the enzyme involved. In this study, the activity, groups and levels of enzyme in gingiva taken from 13 sites of periodontal disease were determined using both biochemical and radioimmunological methods. A linear correlation between the activity and the level of II-$PLA_2$ was observed. No I-$PLA_2$ was found in any of the samples tested. These data suggest that the $PLA_2$ activity found in the GCF of patients with periodontal disease does not belong to the I-$PLA_2$ but to the II-$PLA_2$ group.**

**Key words**: Disease activity, Periodontal disease, Phospholipase $A_2$

### Introduction

Intracellular phospholipase $A_2$ ($PLA_2$; EC 3.1.1.4) catalyses the hydrolysis of the ester bound at the *sn*-2 position of glycerophospholipids and is a key enzyme in the production of potent inflammatory mediators, including prostaglandins, leukotrienes and platelet activating factor. Recently, it has been demonstrated that mammalian $PLA_2$ can be classified into at least three groups on the basis of their primary structure.[1] Two of these groups are designated I- and II-$PLA_2$, and are found in pancreatic juice and a variety of tissues other than pancreas, respectively. Both groups have a molecular mass of 14 kDa.[2] The third group is present in the cytosolic fraction of various tissues and has a molecular weight of 85 kDa.[3] Among these enzyme groups, only II-$PLA_2$ has been considered to play an important role in the process of inflammation, for example, in rheumatoid arthritis[4] or inflammatory bowel disease.[5] In our previous studies, it was demonstrated that rat gingival cells had II-$PLA_2$ activity and that this activity was induced by the inflammatory cytokines interleukin-1$\beta$ (IL-1$\beta$) and tumour necrosis factor-$\alpha$ (TNF-$\alpha$), but inhibited by the anti-inflammatory cytokine transforming growth factor-$\beta$ (TGF-$\beta$).[6,7] In addition, we also demonstrated $PLA_2$ activity in human gingival crevicular fluid (GCF) from patients with periodontal disease and indicated that this activity appeared to reflect periodontal disease conditions (active or resting), suggesting that its potential usefulness as a marker for the assessment of disease activity.[8] However, the maximum volume of GCF was too small to determine whether the activity if contained belonged to the II-$PLA_2$ group, even with the sensitive method we employed. Since GCF contains infiltrates from adjacent gingival tissue that enter the gingival crevice, the enzyme activity in GCF reflects that in the gingival tissue.

To clarify the type of $PLA_2$ present in GCF from patients with periodontal disease, the activity and level of $PLA_2$ were measured in the gingival tissue using both biochemical and radioimmunological assay systems, respectively.

### Materials and Methods

*Gingival tissue:* Gingival tissues from seven patients (mean age 42.0 ± 2.2 years) who had at least five teeth with a pocket depth of more than 6 mm were used. The depth of the periodontal pocket (gingival crevice), which usually indicates the degree of periodontal tissue destruction, and its measurement is considered to be the most reliable tool for the diagnosis and assessment of periodontal disease. A tooth with healthy periodontal tissue has a pocket depth of less than 2 mm, whereas a tooth with a periodontal pocket depth of more than 4 mm is considered to be affected by periodontal disease. None of the patients were receiving or had previously received any medication or periodontal treatment before the study.

*Gingival tissue preparation:* Inflammatory gingival tissues were taken from 13 sites in the seven patients at the time of periodontal surgical therapy. The

© 1995 Rapid Communications of Oxford Ltd excised tissues were rinsed thoroughly with ice-cold isotonic solution and weighed. Then they were minced and homogenized in 100 mM Tris-HCl (pH 7.4) using a glass homogenizer at 2 000 rpm for 30 s, followed by centrifugation at 20 000 × **g**. An aliquot of each supernatant was used as the enzyme source for either biochemical determination of $PLA_2$ activity or radioimmunological $PLA_2$ assay.

*Biochemical assay for $PLA_2$ activity:* Phospholipase $A_2$ activity was determined according to the method described by Tojo *et al.*[9] using 0.8 mM 1-palmitoyl-2 oleoyl-*sn*-glycero-3-phosphoglycerol as a substrate in the presence of 5 mM cholate. Fatty acids released by $PLA_2$ were labelled with 9-anthryldiazomethane and then separated by high-performance liquid chromatography column, followed by quantitation using marganic acid as an internal standard. Calcium-dependent $PLA_2$ was estimated as the difference between the activity assayed in the presence of 5 mM $CaCl_2$ and that in the presence of 10 mM ethylenediaminetetraacetate.

*Radioimmunoassay for I- and II-$PLA_2$:* The level of I- and II-$PLA_2$ in the supernatant was measured using a radioimmunoassay kit (Shionogi Pharmaceutical Ltd, Osaka, Japan) and a monoclonal antibody against human pancreatic group I- or splenic II-$PLA_2$ as described elsewhere.[10]

### Results and Discussion

Figure 1 shows the sensitivity of the RIA used in this study and representative II-$PLA_2$ levels as a function of wet weight of gingival tissue. As can be seen, a minimum gingival tissue wet weight of about 1 mg was enough for detection of II-$PLA_2$. The level of II-$PLA_2$ increased with gingival wet weight over a range of 1 to 25 mg in a dose-dependent manner. It was not possible to determine whether the activity belonged to I- or II-$PLA_2$ in GCF, because usually the volume of GCF is less than 3 μl, which is insufficient for RIA. However, the results suggested that the $PLA_2$ activity detected in GCF was classifiable by examining biopsy samples of gingival tissue. It would be acceptable and meaningful not only for periodontologists but also for patients for about 1 mg wet weight of gingiva to be taken as a biopsy sample to obtain precise and predictable information on the diseased sites.

Table 1 shows the levels of $PLA_2$ determined by the RIA in each supernatant of gingival tissue from 13 different sites. The mean level of II-$PLA_2$ was 83.7 pg/mg wet weight, with wide ranges in the enzyme level from 5 to 216 pg/mg wet weight. This wide range of values was not surprising, because our previous data showed that $PLA_2$ activity was well correlated only with periodontal disease activity, and not with the degree of destruction caused by the previous disease

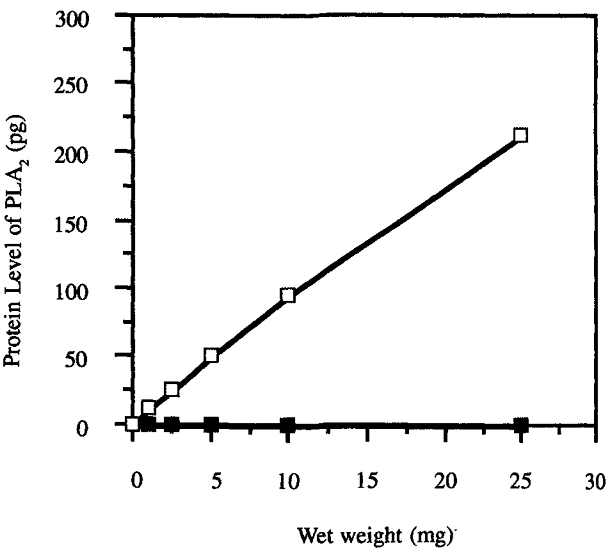

FIG. 1. Levels of group I- and II-phospholipase $A_2$ ($PLA_2$) as a function of wet weight of gingival tissue. Gingival tissue from a patient with periodontal disease was taken at the time of surgical therapy. The level of II-$PLA_2$ increased with the increase of tissue weight, but no I-$PLA_2$ was found.

activity.[8] It has also been shown that II-$PLA_2$ activities found in inflamed sites other than those due to periodontitis, such as rheumatoid arthritis,[4] Crohn's disease[5] and septic shock,[11] are well correlated with disease activity, and thus II-$PLA_2$ is believed to play an important role in the initiation and propagation of the inflammatory process in these diseases. Recently, we showed that GCF sampled from sites of active periodontitis had higher $PLA_2$ activity than those from quiescent sites, although we could not identify whether the activity belonged to group I or II.[8] These activities detected in GCF were thought to be those induced in the inflamed gingival tissues followed by secretion into the gingival crevice. In contrast, none of the samples tested had any detectable levels of I-

**Table 1**. The levels of I-$PLA_2$ and II-$PLA_2$

| Site | $PLA_2$ (pg/mg wet weight) | |
|---|---|---|
| | Group I | Group II |
| 1 | n.d. | 12 |
| 2 | n.d. | 169 |
| 3 | n.d. | 120 |
| 4 | n.d. | 88 |
| 5 | n.d. | 5 |
| 6 | n.d. | 206 |
| 7 | n.d. | 216 |
| 8 | n.d. | 131 |
| 9 | n.d. | 6 |
| 10 | n.d. | 14 |
| 11 | n.d. | 87 |
| 12 | n.d. | 17 |
| 13 | n.d. | 17 |

Levels of I-$PLA_2$ and II-$PLA_2$ were determined by RIA. A wide range of II-$PLA_2$ levels was observed in gingival tissues, but no I-$PLA_2$ was found in any of the samples tested. n.d., not detectable.

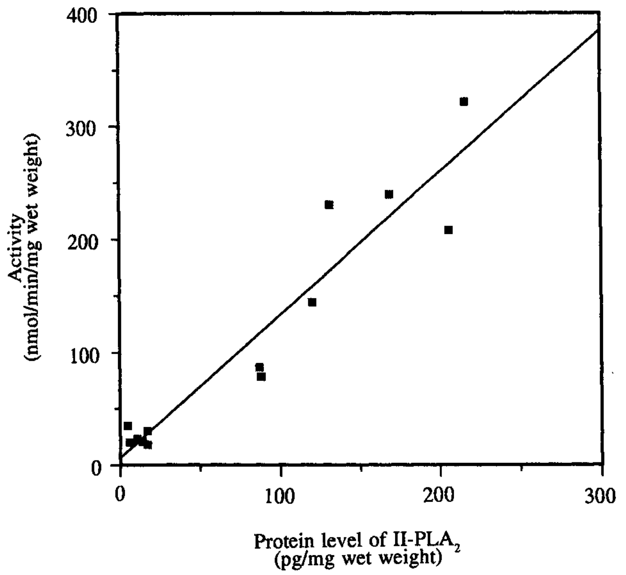

FIG. 2. Relationship between the activity and level of II-$PLA_2$. There is a linear correlation between II-$PLA_2$ activity and level ($r$ = 0.907, Pearson product moment correlation coefficients).

$PLA_2$ using this assay system. These results suggest that the $PLA_2$ activity found in the GCF of patients with periodontal disease is not I-$PLA_2$ but II-$PLA_2$.

It has been reported that serum contains many factors that modulate the activity of $PLA_2$, for example, an activating protein such as $PLA_2$-activating protein[12] and an inhibitory protein such as C3dg.[13] Up to now it has not been clarified whether the elevated $PLA_2$ activity in GCF and gingival tissue is due to an increase in enzyme concentration or to direct activation of the enzyme. Figure 2 shows the relationship between the level of II-$PLA_2$ determined by RIA and the activity determined by biochemical assay in the 13 samples. As can be seen, a highly significant correlation ($r$ = 0.907, $p < 0.001$) between the level and activity of II-$PLA_2$ was observed. These findings indicate that the $PLA_2$ activity detected in GCF was attributable primarily to an increase in the protein concentration of II-$PLA_2$, probably via *de novo* protein synthesis in the gingiva, and not to activation of the enzyme. This close correlation between the level and activity of II-$PLA_2$ agrees well with previous reports on septic shock,[11] malaria,[14] or Crohn's disease and ulcerative colitis.[15]

We showed that inflammatory cytokines such as IL-1β and TNF-α induced, whereas an anti-inflammatory cytokine, TFG-β, inhibited the synthesis and secretion of II-$PLA_2$ in cultured rat gingival fibroblastic cells.[6,7] It is well known that these cytokines are present in GCF and levels of IL-1β in gingival tissue correlate well with disease activity of periodontitis.[16] Although it was not possible to identify the source of II-$PLA_2$ in gingival tissue from this study, it is considered that gingival cells in the inflamed sites are one of the most likely candidates.

In summary, the present results suggest that $PLA_2$ activity detected in GCF does not belong to I-$PLA_2$, but to II-$PLA_2$. It is also suggested that measurement of either the level of II-$PLA_2$ in biopsy samples of inflamed gingiva or the enzyme activity in GCF may be useful for detecting sites of active disease in periodontitis.

### References

1. Van den Boch H. Intracellular phospholipase A. *Biochem Biophys Acta* 1980; **607**: 191–246.
2. Heinrikson RL, Krueger ET, Keim PS. Amino acid sequence of phospholipase $A_2$-α from the venom of *Crotalus adamanteus*. A new classification of phospholipase $A_2$ based upon structural determinants. *J Biol Chem* 1977; **252**: 4913–4921.
3. Murakami M, Kudo I, Umeda M, *et al.* Detection of three distinct phospholipase $A_2$ in cultured mast cells. *J Biochem* 1992; **111**: 175–181.
4. Pruzanski W, Keystone EC, Sternby B, Bombardier C, Snow KM, Vadas P. Serum phospholipase $A_2$ correlates with disease activity in rheumatoid arthritis. *J Rheumatol* 1988; **15**: 1351–1355.
5. Minami T, Tojo H, Shinomura Y, Tarui S, Okamoto M. Raised serum activity of phospholipase $A_2$ immunochemically related to group II enzyme in inflammatory bowel disease; its correlation with disease activity of Crohn's disease and ulcerative colitis. *Gut* 1992; **33**: 914–921.
6. Shinohara H, Amabe Y, Komatubara T, *et al.* Group II phospholipase $A_2$ induced by interleukin-1b in cultured rat gingival fibroblasts. *FEBS Lett* 1992; **304**: 69–72.
7. Ishida H, Shinohara H, Amabe Y, Tojo H, Nagata T, Wakano Y. Effects of interleukin 1β, tumor necrosis factor α and transforming growth factor β on group II phospholipase $A_2$ activity in rat gingival fibroblasts. *J Periodont Res* 1993; **28**: 517–520.
8. Ishida H, Shinohara H, Nagata T, Nishikawa S, Wakano Y. Phospholipase $A_2$ activity in gingival crevicular fluid from patients with periodontal disease: a possible marker of disease activity. *Mediators of Inflammation* 1994; **3**: 17–21.
9. Tojo H, Ono T, Okamoto M. Spleen phospholipase $A_2$. *Methods Enzymol* 1991; **197**: 390–399.
10. Masuda Y, Ogawa M, Sakamoto K, *et al.* Development of a radioimmunoassay for human group-II phospholipase $A_2$ and demonstration of postoperative elevation. *Enzyme* 1991; **45**: 200–208.
11. Vadas P, Scott K, Smith G, *et al.* Serum phospholipase $A_2$ enzyme activity and immunoreactivity in a prospective analysis of patients with septic shock. *Life Sci* 1992; **50**: 807–811.
12. Bomalaski JS, Fallon M, Turner RA, Crooke ST, Meunier PC, Clark MA. Identification and isolation of a phospholipase A2 activating protein in human rheumatoid arthritis synovial fluid: induction of eicosanoid synthesis and an inflammatory response in joints injected *in vivo*. *J Lab Clin Med* 1990; **116**: 814–824.
13. Suwa Y, Kudo I, Imaizumi A, *et al.* Proteinaceous inhibitors of phospholipase $A_2$ purified from inflammatory sites in rats. *Proc Natl Acad Sci USA* 1990; **86**: 2395–2399.
14. Vadas P, Keystone J, Stefanski E, Scott K, Pruzanski W. Induction of circulating group II phospholipase $A_2$ expression in adults with malaria. *Infect Immun* 1992; **60**: 3928–3931.
15. Minami T, Tojo H, Shinomura Y, Komatubara T, Matsuzawa Y, Okamoto M. Elevation of phospholipase $A_2$ protein in sera of patients with Crohn's disease and ulcerative colitis. *Am J Gastroenterol* 1993; **88**: 1076–1080.
16. Stashenko P, Fujiyoshi P, Obernesser MS, Prostak L, Haffajee AD, Socransky SS. Levels of interleukin 1β in tissue from sites of active periodontal disease. *J Clin Periodontal* 1991; **18**: 548–554.

**Received 1 November 1994; accepted 9 November 1994**
